# Management of Anterior Mediastinal Hodgkin’s Lymphoma in Polycystic Kidney Disease: A Case Report

**DOI:** 10.7759/cureus.81583

**Published:** 2025-04-01

**Authors:** Andrea N Wang, Emily Heath, A. Rashid Dar, Keng Yeow Tay, Louise Moist

**Affiliations:** 1 Radiation Oncology, Schulich School of Medicine & Dentistry, Western University, London, CAN; 2 Surgery, Cumming School of Medicine, University of Calgary, Calgary, CAN; 3 Radiology, Victoria Hospital, London, CAN; 4 Radiology, London Health Sciences Centre, London, CAN; 5 Medicine, Schulich School of Medicine & Dentistry, Western University, London, CAN

**Keywords:** chemoradiation, chronic kidney disease (ckd), hodgkin’s lymphoma, polycystic kidney disease (pkd), superior vena cava (svc) syndrome

## Abstract

This case report presents a 33-year-old male with polycystic kidney disease (PKD) who presented with superior vena cava syndrome due to an anterior mediastinal mass. Imaging and pathology confirmed a Hodgkin’s lymphoma of nodular sclerosis subtype of the mediastinum. Despite chronic kidney disease, prioritization to treat the Hodgkin’s lymphoma was taken with careful monitoring of renal function. Renal function declined throughout chemotherapy, necessitating modification to the chemotherapy regimen, but not requiring dialysis. Three years following remission from lymphoma, renal function continued to decline, prompting renal transplant and bilateral nephrectomy. This case highlights considerations in treatment for oncological disease with pre-existing decreased kidney function.

## Introduction

Advanced chronic kidney disease is often a limiting factor in determining appropriate treatment in people with cancer. We present a case of a patient with newly diagnosed Hodgkin’s lymphoma, a cancer of the lymphatic cells, in the anterior mediastinum. Management was complicated by the presence of advanced chronic kidney disease secondary to polycystic kidney disease (PKD). We highlight the special considerations and monitoring of kidney function and the side effects of chemotherapy on the heart and lungs, which were necessary for long-term survival.

## Case presentation

A 33-year-old man presented with a swollen neck and face and an enlarged lymph node in the posterior cervical region, around the size of a golf ball. Relevant past medical history included PKD since the age of 19 and secondary hypertension (heart rate of 72, blood pressure of 164/90 mmHg) that was managed with 12.5 mg hydrochlorothiazide daily, with stable creatinine ranging between 130 and 140 µmol/L. The patient also previously had an episode of facial flushing, which was thought to be due to the use of an angiotensin-converting enzyme inhibitor; this settled down after switching to angiotensin II receptor blockers for hypertension management.

Investigations included a chest X-ray, which revealed a widening of the upper mediastinum. Subsequent computed tomography (CT) scans showed a large left anterior mediastinal mass measuring 6.5 x 10 cm, with extension into the right paratracheal region, and nodes measuring up to 2.0-2.5 cm. The swollen neck and face were clinically correlated with superior vena cava compression, with the formation of collaterals. CT scans also showed mild pericardial effusion, while the lungs were otherwise normal. Given the presentation of a mediastinal mass, the differentiation between lymphoma, metastatic germ cell tumor, and thymoma required further investigation. Serum markers were negative for alpha-fetoprotein and human chorionic gonadotropin. A transthoracic core needle biopsy was inconclusive, and CT-guided excisional nodal biopsies were negative. The patient was partially treated with dexamethasone and prednisone for three weeks. On prednisone, the neck lesion disappeared, and a repeat CT scan showed that the mediastinal mass appeared to have shrunken down to 4.5 x 6.5 cm. The anterior mediastinal mass was biopsied with a mediastinoscopy. Morphology and immunohistochemical stains confirmed that the patient had stage IIb classic Hodgkin’s lymphoma of nodular sclerosis subtype (CD15+, CD30+, CD20-, CD5-, and Epstein-Barr virus-).

The patient had previous documentation on ultrasound of bilateral polycystic kidneys and noticed an increase in voiding frequency over the last 1.5 months prior to admission. He reported getting up to urinate four times a night and occasionally had hematuria. A CT scan of the abdomen revealed that both kidneys were massive (at least 20 cm in size) with multiple round low-density cysts. Some of these cysts were hyperdense, and there was a 5 mm calcification in the lower pole of the left kidney. There was no evidence of hepatosplenomegaly or inguinal lymphadenopathy. Contrast was avoided in the CT scan due to elevated creatinine at 143 µmol/L.

Lymphoma treatment and follow-up

Lymphoma management was complicated by superior vena cava obstruction and PKD, requiring careful consideration and monitoring of the cardiac and renal side effects of chemotherapy. Throughout the treatment routine, complete blood count, platelets, urea, and creatinine were monitored in case of need for dialysis. The patient was treated with doxorubicin 46 mg, bleomycin 18.4 units, vinblastine 11 mg intravenous, and dacarbazine 688 mg intravenous (AVBD chemotherapy).

While in the hospital, the patient became hypertensive with a blood pressure (BP) of 190/110 mmHg, so losartan 50 mg daily was prescribed, and hydrochlorothiazide was increased to 25 mg daily. The patient’s creatinine reached 170 µmol/L from 148 µmol/L, which was noted to be an expected increase. There was uncertainty surrounding whether the patient’s increased levels of urea and creatinine were secondary to chemotherapy treatment or a remote effect of PKD. The dose of bleomycin was subsequently decreased to 15.6 units, or 75% of the original dose. Considering the patient’s diagnosis, his clinical symptoms were slow to show improvement. Radiation therapy was initiated three weeks following the administration of cycle 2A of AVBD chemotherapy. The patient received radiation to the mediastinum and neck for a total of 3000 cGy isocenter dose for over four weeks in 20 fractions. Routine anti-emetics were used for nausea and vomiting. The patient tolerated radiation without interruption. AVBD chemotherapy was resumed nearly four months later.

**Figure 1:**
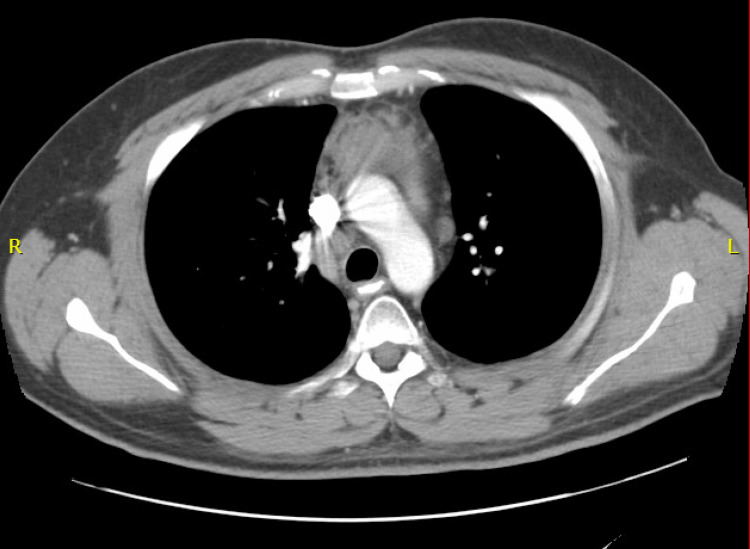
Anterior mediastinal mass anterior to the aortic arch, measuring 3.8 x 5.1 cm, essentially unchanged from the start of chemotherapy.

During the course of his chemotherapy, the patient presented with a productive cough and shortness of breath, which were presumed to be due to bleomycin toxicity. Bleomycin was subsequently excluded from chemotherapy treatments from cycle 4A thereafter. Following cycle 4B of chemotherapy, the patient was admitted for neutropenia fever (leukocytes <0.5 x 109 cells/L). CT of the thorax suggested no significant change in the size of the anterior mediastinal mass (Figure 1), but consolidation in air bronchograms was consistent with pneumonia. He was treated empirically with imipenem prior to negative blood culture results and changed to levofloxacin two days later.

Following the completion of the 6A cycle of AVBD chemotherapy, the patient entered remission from lymphoma with a CT thorax revealing the mediastinal mass as 3.5 x 2.8 cm. During chemotherapy, the patient experienced an acute deterioration in renal function with increased levels of creatinine (>200 µmol/L). This was presumed to be due to his history of PKD since he was not receiving nephrotoxic agents, and all radiation was done above the diaphragm.

Renal decline and treatment

**Figure 2:**
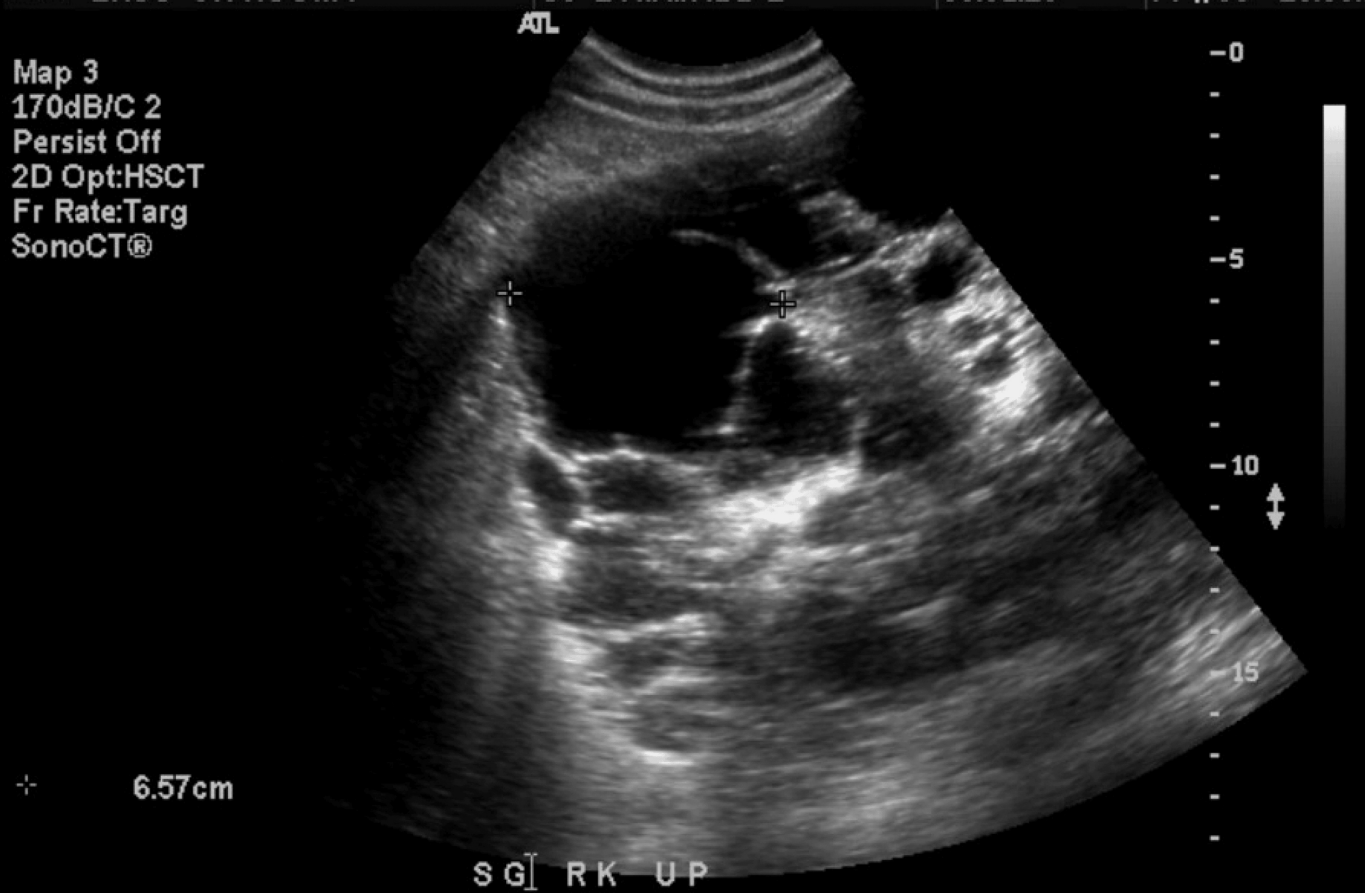
Polycystic kidney disease on ultrasound in the right kidney with a large cyst measuring 6.6 cm.

Three years following remission from lymphoma, kidney function continued to decrease. Despite normal BP at 138/96 mmHg, creatinine was 418 µmol/L, increasing approximately 50 µmol/L per year since lymphoma remission. An ultrasound revealed bilateral enlarged kidneys with innumerable cysts with the largest in the right upper pole measuring 6.6 cm (Figure 2) and the left upper pole measuring 6.9 cm. The patient elected to have a pre-emptive standard donor renal transplant for this end-stage renal disease secondary to PKD. The patient never required dialysis. He eventually also had bilateral nephrectomy of non-functioning kidneys, and his creatinine, kidney function, and blood pressure remained well-controlled. We report that after entering remission, the patient has not presented with any further evidence of lymphadenopathy or malignancy in the thorax. The patient also continues to be followed for his renal function, which has remained well over the following 18 years.

## Discussion

PKD is a disorder characterized by the formation of multiple fluid-filled cysts in the kidneys [1]. It is estimated to affect 8 million individuals worldwide, and autosomal dominant mutations are the most common genetic etiology for PKD [1]. The most common autosomal dominant mutations for PKD are *PKD1* and *PKD2* [2,3]. Cysts are fluid-filled and contribute to a bumpy contour and expanding pressure within the kidney, which can lead to several complications, including hypertension and related cardiac dysfunction, kidney and urinary infection, stones, and eventually end-stage renal disease [4]. PKD accounts for approximately 5% of all cases of end-stage renal disease in the United States. PKD is also associated with the formation of cysts in other organs, most commonly in the liver, where 60% of PKD cases have shown polycystic liver disease [5]. On average, PKD patients have a life expectancy that ranges between 53 and 70 years, depending on the subtype of the disease [6]. While PKD is characterized by decreased eGFR [7], some studies have also shown normal serum creatinine levels in PKD patients [8,9]. Estimation of renal function in PKD patients has shown some inaccuracies, and reliable methods of measurement are still under discussion [10].

Lymphomas of the mediastinum may be systemic or of primary etiology, the latter being rare and only making up 1% of cases [11-14]. Primary mediastinal lymphomas are defined as involvement by lymphoma of mediastinal lymph nodes, the thymus, or both, without extranodal or systemic disease at presentation [15]. An accurate estimation of its frequency is difficult due to its rarity and the limited number of studies [15]. Interestingly, genetic mutations and immunosuppression may predispose patients to developing lymphomas. This was previously demonstrated in a case where non-Hodgkin’s lymphoma occurred 12 years following transplantation in autosomal dominant PKD [16]. However, these cases of lymphoma have only been described in the context of colon, liver, and kidney cancer [17].

In this case, consideration to treat the anterior mediastinal mass was complicated by potential declining renal function due to the patient’s pre-existing history of PKD. While literature was scant to guide treatment, urgent intervention of the anterior mediastinal Hodgkin’s lymphoma was required due to superior vena cava syndrome. The decision was made to prioritize the treatment of the Hodgkin’s lymphoma with thorough documentation of the patient’s renal function throughout chemoradiation therapy. While receiving chemotherapy, declining renal function prompted modification to the chemotherapy regimen, which was successful in reaching remission. There was no requirement for dialysis throughout the treatment of lymphoma; however, the patient would eventually require a renal transplant and bilateral nephrectomy due to enlarged non-functioning kidneys. It is unclear whether the patient’s renal function declined due to the nature of PKD or due to chemoradiation, as creatinine levels remained steadily high at levels ranging between 143 and 236 µmol/L during chemoradiation. Of note, as radiation therapy was limited to the mediastinum, it was unlikely to have been the primary cause of renal decline. Previous studies have demonstrated lymphoma to be associated with acute kidney injury, with independent predictors, including sepsis and aminoglycosides, among others. In particular, rituximab, cyclophosphamide, vincristine, and prednisolone (R-CVP) chemotherapy use in lymphoma has been associated with acute kidney injury [18]. Other studies have shown that the addition of targeted drugs such as brentuximab vedotin would benefit intensive chemotherapy regimens [19].

## Conclusions

This case report highlights multiple considerations for oncologic patients with pre-existing PKD. Due to pre-existing elevated creatinine levels, contrast in CT imaging was avoided. Increasing creatinine levels during chemotherapy necessitated modifications to bleomycin dosing, and symptoms of bleomycin toxicity would prompt its exclusion from further therapy. Dialysis was not required throughout the treatment of lymphoma, and the patient entered remission following chemoradiation. However, the patient’s renal function eventually declined three years following remission, which required renal transplantation. While there is still uncertainty surrounding whether the decline in renal function was due to chemotherapy or due to the clinical course of PKD, this case demonstrates the considerations taken to preserve kidney function and chemotherapy side effects. Our case report showed excellent outcomes in the treatment of anterior mediastinal Hodgkin’s lymphoma in the presence of chronic kidney disease secondary to PKD.
